# Digital Adherence Technologies and Differentiated Care for Tuberculosis Treatment and Their Acceptability Among Persons With Tuberculosis, Health Care Workers, and Key Informants in the Philippines: Qualitative Interview Study

**DOI:** 10.2196/54117

**Published:** 2024-07-23

**Authors:** Chung Lam Leung, Jason Alacapa, Bianca Gonçalves Tasca, Andre Daniel Villanueva, Saniata Masulit, Marvin Louie Ignacio, Kathleen Nicole Uy, Christopher Pell, Kristian van Kalmthout, Rachel Powers, Katherine Fielding, Degu Jerene

**Affiliations:** 1 KNCV Tuberculosis Foundation Den Haag Netherlands; 2 KNCV Tuberculosis Foundation Philippines Metro Manila Philippines; 3 TB Centre and Department of Infectious Disease Epidemiology Faculty of Epidemiology and Population Health London School of Hygiene & Tropical Medicine London United Kingdom

**Keywords:** tuberculosis, digital adherence technologies, implementation, acceptability, qualitative research, Philippines, digital health, tuberculosis treatment, support strategy, support, medication adherence, health care workers, interview, interviews, user, user privacy, privacy, digital adherence

## Abstract

**Background:**

Digital adherence technologies (DATs) are being studied to determine their potential to support tuberculosis (TB) treatment and address the shortcomings of directly observed therapy. Previous research has shown inconclusive results on whether DATs can enhance medication adherence among persons with TB.

**Objective:**

This study aims to understand the acceptability of DATs, namely, medication labels and smart pillboxes, among persons with TB, health care workers (HCWs), and key informants (KIs) in the Philippines. The objective is to gain valuable insights that can inform the design and implementation of DATs in the Southeast Asian region, which meet the needs and preferences of end users.

**Methods:**

Persons with TB, HCWs, and KIs were recruited from intervention facilities to participate in in-depth interviews conducted between March 2022 and January 2023. These interviews were transcribed and translated into English. A thematic analysis was carried out using NVivo software (Lumivero) to identify and analyze themes. Themes were then structured within a modified social-ecological model.

**Results:**

A total of 25 persons with drug-sensitive TB and 20 HCWs or KIs were interviewed. Both groups emphasized that users’ technology literacy level, financial conditions, and motivation to be cured determined how they interacted with the DAT. They also acknowledged that DATs helped foster their relationship with HCWs and enabled efficient treatment support. Concerning technology, persons with TB found DATs easy to use and able to reduce clinic visits. HCWs mentioned that DATs added to their workload but also allowed them to support users who missed doses. However, both groups experienced technical challenges with DATs. Regarding program implementation, users appreciated the clear explanations and demonstrations provided by HCWs. Yet, some users reported inconsistencies between DAT settings and the information provided. HCWs stressed the importance of comprehensive training and sufficient resources for effective program implementation in the future. At the community level, both groups noted that DATs and program design protected users’ privacy and reduced the risk of stigma. Finally, users and HCWs shared various contextual factors that influenced their experience with DAT, including infrastructure challenges and the impact of the COVID-19 pandemic.

**Conclusions:**

In the Philippines, persons with TB and HCWs showed a high level of acceptance and satisfaction with the impact of DAT and program design. They expressed a desire for the continuation of DATs. The challenges encountered underscore the need for ongoing technological development to minimize malfunctions, enhance the capacity of health facilities, and improve infrastructure. DATs have demonstrated their ability to strengthen user-HCW relationships and protect users from stigmatization. Additional efforts are required to scale up the DAT program in the Philippines.

## Introduction

Endorsed by the World Health Organization (WHO), directly observed therapy (DOT) is part of tuberculosis (TB) programs worldwide at varying degrees of implementation. Despite its widespread implementation, shortcomings in the DOT approach have emerged. Frequent health facility visits disrupt patients’ daily routines, increase financial burdens, and impact patient autonomy [1,2]. Hence, alternative treatment support strategies such as digital adherence technologies (DATs) are being evaluated to determine their effectiveness in supporting TB treatment and addressing the limitations of DOT [3-5]. These technologies leverage mobile phones, computers, web-based platforms, and smart pillboxes to collect real-time data on patient treatment adherence.

Studies conducted in Kenya, Vietnam, Peru, and China have shown that DATs have the potential to improve medication adherence among persons with TB [4,6-9]. By empowering persons with TB to take a more active role in managing their health conditions alongside health care providers, these technologies have the potential to reduce the financial burden and disruption associated with DOT and promote autonomy [10]. Additionally, DATs allow health care workers (HCWs) to identify individuals who may require additional support during their treatment [11]. Studies conducted elsewhere have yielded inconsistent evidence regarding the impact of DATs on improving adherence outcomes compared to the standard of care, which includes DOT [4,12-17].

The multicountry Adherence Support Coalition to End TB (ASCENT) project was initiated to add to the evidence base around DATs [18]. This multidisciplinary project compared the impact of DATs against the standard of care in terms of treatment outcomes and sought to determine the feasibility and acceptability of DATs for TB treatment support. Understanding the acceptability of DATs among persons with TB and HCWs implementing DATs are key to determining the technology’s long-term impact and viability. According to Sekhon et al [19], the acceptability of a health care intervention is determined by the “people delivering or receiving a health care intervention consider it to be appropriate, based on anticipated or experienced cognitive and emotional responses to the intervention.” Currently, there is limited research examining the acceptability of DATs among persons with TB and HCWs in the Southeast Asian region; further research is needed to determine their long-term impacts and viability [20-22].

Drawing on in-depth interviews conducted as part of the ASCENT study in the Philippines, this paper describes the perceptions and experiences of persons with TB, HCWs, and key informants (KIs) regarding DATs. It seeks to address the evidence gap around the acceptability of DATs and ultimately seeks to garner insights to design and implement DATs in the Southeast Asian region that are responsive to the needs and preferences of the end users.

## Methods

### Study Setting

This study is part of the ASCENT: 4-country evaluation of DATs for TB treatment, aimed at evaluating the effectiveness of DATs across 4 countries [18]. The DAT intervention included the provision of medication labels or smart pillboxes (Multimedia Appendix 1), a digital adherence platform, and differentiated care among adults with drug-sensitive tuberculosis (DS-TB) in the Philippines, South Africa, Tanzania, and Ukraine.

According to the WHO [23], the total TB incidence rate in the Philippines was 638 per 100,000 population in 2022. In the 2000s, directly observed treatment short-course achieved full coverage in the Philippines, being available in public and private health care facilities [24]. As per the National Tuberculosis Control Program (NTP) Manual of Procedures 6th edition of the Philippines [25], DOT can be administered within a health care facility on a daily basis by an HCW or outside the facility by a trained treatment supporter, typically receiving a 1-week supply of medication. The treatment approach is determined jointly by the persons with TB and the health care provider.

In the Philippines, the ASCENT study used a cluster randomized trial, using health facilities as the unit of randomization. In total, 64 health facilities from the Bulacan and Pampanga provinces in Region III of the Philippines were included. Of the 32 facilities in the intervention arm, 16 used medication labels, while the remaining ones implemented smart pillboxes. Adults (18 years or older) starting the DS-TB regimen were invited to enroll. In medication label facilities, DAT was offered to consenting persons with TB who had access to a mobile phone, either their own or that of their treatment companion. Persons with TB who did not have access to or had difficulties using mobile phones were offered a smart pillbox while supplies lasted in that facility. In smart pillbox facilities, consenting persons with TB were offered a smart pillbox. Individuals younger than 18 years and with drug-resistant TB were excluded from the study according to the study protocol [18].

The overarching digital adherence platform was used to track adherence information for both DATs. Medication label users were asked to take their daily medication and then log their intake by sending an SMS text message using the toll-free number on the medication blister packs. Some telecom operators in the Philippines require a positive credit balance (Php 1.00 or approximately US $0.02) to send free SMS text messages. Smart pillbox users received a pillbox to store their medication. When the user opens the box to take their medication, the box’s sensor sends a notification to the platform registering the medication intake. In cases of an unstable network connection, the box records the opening and transmits the timestamped data once the network is available again. If a user fails to send a code or open the box before 4 PM, the system triggers a reminder message sent to the user’s phone to prompt the patient to take and log their daily dose. If no dose is subsequently logged before midnight, the system triggers a notification message sent to the user at 8 AM the following morning, alerting them of their missed logged dose and reminding them to complete the current day’s intake.

The digital adherence platform allows HCWs to remotely view users’ treatment adherence. Each person with TB is linked to an adherence calendar, visualized by 1 colored box per day. Individuals who have logged their dose by opening their smart pillbox or sending a message will have that day marked in green on the platform, whereas those who have missed a dose will have it marked in red. If a person with TB missed 1 daily dose, the HCW was expected to make a follow-up call to provide support. If a person with TB missed doses for 2 consecutive days, community-level HCWs were expected to visit the user. Persons with TB were only required to visit the facility to refill their medication or seek medication advice. HCWs were expected to check the system daily, wherein they viewed the adherence calendars and accessed Task Lists—consolidated lists per support action (eg, call and home visit) indicating which persons with TB required follow-up support. This full pathway of support escalation, or differentiated care, was determined together by in-country health system stakeholders, implementers, and patient advocacy groups.

User adherence records were captured by the adherence platform and then transferred to the local Department of Health (DoH) Integrated Tuberculosis Information System. Prior to the implementation, training was provided to all HCWs from the 32 intervention facilities involved in the project on platform use, patient orientation, knowledge about DATs, and treatment counseling.

### Participant Recruitment and Data Collection

We used semistructured, in-depth interview methods. DAT users (medication label and smart pillbox), HCWs, and KIs were purposively recruited from up to 10 ASCENT DAT facilities in the Philippines. HCWs and KIs were selected based on their role and level of involvement in the project. Interviews were conducted in enclosed meeting rooms within the facilities, and before the interviews, all participants provided informed consent for the interview and it being recorded. Data collection took place between March 2022 and January 2023.

For user interviews, we aimed to recruit a minimum of 20 participants, comprising 10 male and 10 female participants, who were currently undergoing TB treatment in DAT facilities (offering either DAT). Users were selected based on either demonstrating documented good adherence, receiving differentiated care, or having chosen not to use DAT. Furthermore, we sought to purposively recruit a minimum of 20 DAT HCWs and KIs from the DoH or the NTP of the Philippines.

### Data Collection

The interviews were conducted by 1 researcher and 2 research assistants from the ASCENT Philippines team. These individuals received training in qualitative research methodologies prior to data collection. An open-ended interview guide was used to facilitate the interview with persons with TB. The guide covered topics, such as users’ experiences with using DATs, including unintentional disclosure, factors that facilitated adherence or nonadherence to the treatment, and cultural or other barriers that deterred them from using DATs. Due to COVID-19 restrictions in the Philippines, interviews were conducted via phone calls. Due to language barriers and age considerations, some interviews were conducted in the presence of the user’s treatment companion. The duration of the interviews ranged from 30 to 50 minutes. The same research team used a separate interview guide developed for HCWs and KIs. They were queried about their perceptions and the feasibility of implementing DATs in the Philippines. The duration of these interviews ranged from 40 to 60 minutes. Interviews were conducted in either Filipino or English, depending on the participants’ preference, and were audio recorded.

### Data Processing and Analysis

The recorded interviews were transcribed verbatim in the local language and subsequently translated into English. To ensure accuracy and quality, an independent staff member translated the English transcript back into Filipino for cross-checking. This process helped validate the quality of the translation and ensure that the original meaning was preserved.

Thematic analysis was conducted to identify and analyze themes emerging from interviews, capturing the richness of the data while remaining independent of existing theory [26]. Using critical realism, this analysis describes participants’ experiences with DAT and their interpretation of those experiences in relation to the local context [27].

A total of 2 coders, CLL and BGT, based in the Netherlands, independently coded the transcripts using NVivo software (Lumivero). Regular meetings were held between the coders and other coinvestigators to discuss the coding process and identify emerging themes. Following the establishment of the initial codebook, the coders collaborated with the interviewers from the Philippines to gain a deeper understanding of the interview process, project implementation, and the contextual factors specific to the country. This collaboration aimed to further refine the codebook.

The coding process was conducted separately, categorizing the type of DAT used by persons with TB or overseen by HCWs. After refining the individual codebook for each DAT, a decision was made to consolidate the analysis of the 2 DATs featured in this study. This decision was supported by the observation of overlapping factors influencing persons with TB and HCWs in their use of the 2 DATs.

**Figure 1:**
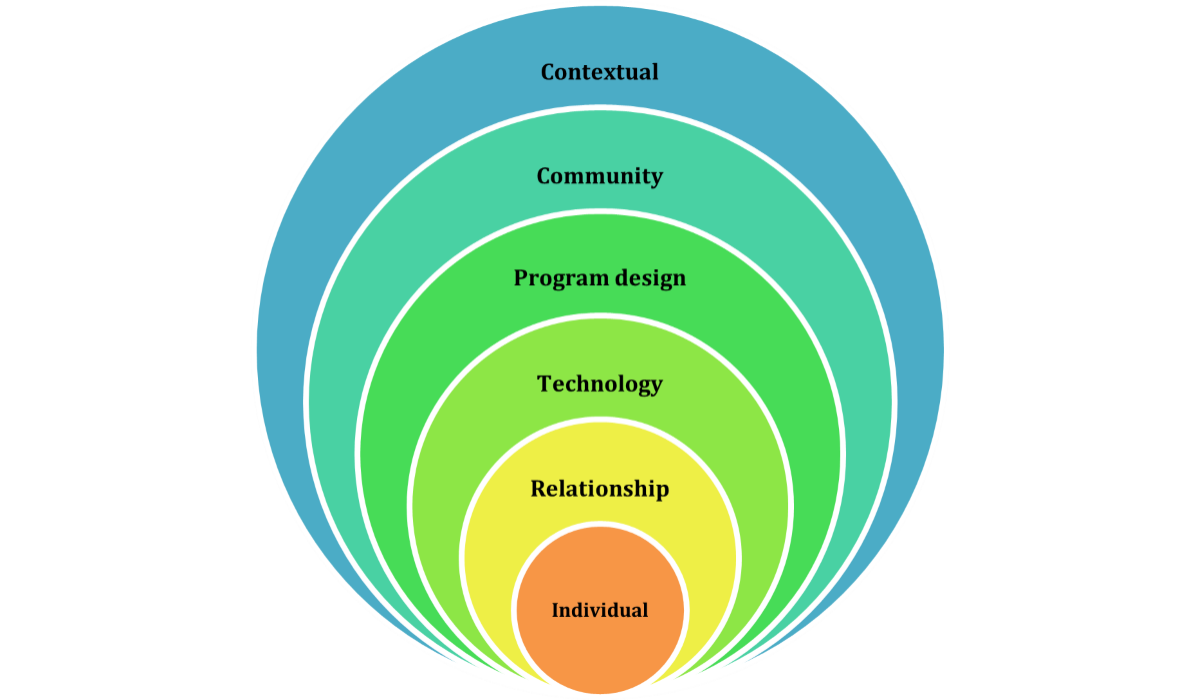
The modified socioecological model (adapted from the findings of Sazzad et al [28] and McLeroy et al [29]).

Following the coding process, the social-ecological model has been modified to present the identified themes into 6 levels (Figure 1). The individual level considers personal factors (eg, age, education, and income) that influence DAT interaction [28,29]. The relationship level focuses on social ties impacting DAT engagement [29]. The technology level explains how the function of DATs impacts user use. Program design levels relate to how the implementation process influences DAT use. The community level encompasses aspects of social culture, norms, and stigma, explaining how these factors guide interactions involving DATs [30]. Finally, the contextual level considers country-specific conditions (eg, infrastructure and policy) affecting DAT implementation.

### Ethical Considerations

The main study has been approved by the WHO Ethical Review Committee (0003296), the Single Joint Research Ethics Board (the Ethics Committee for the Philippines), and the London School of Hygiene & Tropical Medicine Ethics Committee, United Kingdom. Written informed consent was obtained from all participants prior to their interviews. All interview transcripts were deidentified to preserve the privacy of the participants.

## Results

### Characteristics of Participants

Interviews were conducted with 25 persons with DS-TB (12 male and 13 female participants; ages ranged from 22 to 86 years; 14 bacteriologically confirmed TB and 11 were clinically diagnosed) from DAT implementation facilities. Overall, 19 persons used medication labels, and 6 used smart pillboxes. Notably, 2 users who chose not to use DAT were invited to participate in the interview but did not provide consent. As a result, this study included only individuals who used DATs in their treatment.

A total of 20 HCWs and KIs (5 male and 15 female participants) were interviewed. In total, 10 HCWs worked in a medication label facility and 10 in a smart pillbox facility. In terms of professions, 13 of the participants were nurses in health care facilities, 3 were NTP medical coordinators, 2 were midwives, 1 was a medical technologist, and 1 was a data encoder. Due to a shortage of HCWs, midwives were assisting with TB treatment; thus, they were included in this study. The 3 KIs included an NTP medical coordinator, an NTP city health officer from Bulacan, and an NTP municipal health officer from Pampanga. In terms of the geographical distribution of HCWs, 11 were from Bulacan, 8 were from Pampanga, and 1 was from Region III province office (Table 1).

**Table 1:** Interview participant characteristics.

			Values, n (%)
**Persons with TB^a^ (n=25)**			
	**Sex**		
		Male	12 (48)
		Female	13 (52)
	**Age (years)**		
		18-29	7 (28)
		30-39	6 (24)
		40-49	5 (20)
		50-59	3 (12)
		≥60	4 (16)
	**Disease classification**		
		Bacteriologically confirmed	14 (56)
		Clinically diagnosed	11 (44)
	**DAT^b^ type**		
		Medication label	19 (76)
		Smart pillbox	6 (24)
**Health care workers and KIs^c^ (n=20)**			
	**Sex**		
		Male	5 (25)
		Female	15 (75)
	**Role**		
		Nurse	13 (65)
		Midwife	2 (10)
		Medical technologist	1 (5)
		Data encoder	1 (5)
		NTP^d^ medical coordinator	3 (15)
	**Facility**		
		Medication label	10 (50)
		Smart pillbox	10 (50)
	**Province**		
		Bulacan	11 (55)
		Pampanga	8 (40)
		Other	1 (5)

^a^TB: tuberculosis.

^b^DAT: digital adherence technology.

^c^KI: key informant.

^d^NTP: National Tuberculosis Control Program.

### DAT User and HCW Perceptions and Experiences

#### Individual Level

At the individual level, users’ technology literacy, financial conditions, and motivation to be cured influenced their interactions with the DAT. Prior to implementation, HCWs expressed concerns about the potential technological complexity of DAT, which could pose usability and adaptability challenges for users.

... it seems like it’s easier for the patient to have face-to-face interaction with the HCW.

Nurse, female

Users with low technology literacy and older people encountered difficulties in sending the code using the mobile phone. Without the assistance of family members or treatment companions, older people struggled to comprehend the instructions for using DAT. HCWs also found it challenging to explain the system to individuals with limited technology literacy.

Interviewer: When Grandma just started taking medicine, did the nurse teach her as well?

Participant: No, just me because she wouldn’t be able to understand the instructions.

Treatment companion of a medication label user, age ≥60 years, female

So, the challenge when we introduce it to the elderly, they feel like they can’t understand, and it’s hard to use. Then they refuse.

Nurse, female

HCWs observed that many potential DAT users lacked access to a phone or had insufficient credit on their SIM cards, causing fewer individuals to use the medication label. Some HCWs working in the medication label facility mentioned that if the facility ran out of pillboxes, eligible users for joining the DAT program would be excluded from the intervention.

That’s when we have a problem when the patient doesn’t have a cell phone and then they don’t have a support system, so we give them a pillbox.

Nurse, female

Despite the above personal circumstances, many users showed a strong intrinsic motivation to be cured, which drove them to embrace the DAT and remain committed to their treatment. Some users even used supplementary methods, such as paper calendars and phone alarms, to stay on track with their treatment.

It was his willingness to get better. That was his main motivation to continue taking the medications given by the nurse or the doctor.

Treatment companion of a medication label user, age ≥60 years, male

#### Relationship Level

Interpersonal relationships shaped how users interacted with DATs, potentially facilitating their use and potentially being transformed by DATs. Many users disclosed their use of DAT to their family members but did not share this information further. This is because, during their initial facility visit, persons with TB in the Philippines are required to bring along a family member as their treatment companion. Multiple participants mentioned that their family members or treatment companions reminded them to take their medication.

Yes, because aside from me, I was also being reminded by my husband.

Smart pillbox user, age 50-59 years, female

Users and HCWs both acknowledged that using the DAT helped foster trust between them. It enabled efficient and less intrusive treatment support.

there is another monitor on their end where they could see if you took your medicine or not. Doesn’t it make you happy and proud to have something like that?

Medication label user, age 30-39 years, female

Yes, it will help me because there are patients who are compliant and we do not supervise them that much. We have built trust with each other.

Nurse, female

I can say that I have become closer to the patients but it really depends on the person. They know that I am checking their adherence, so they can approach me with more ease.

Nurse, female

#### Technology Level

At the technology level, both groups experienced positive effects and technical challenges of the DAT that affected TB care. Among the positive effects, many DAT users reported that it reduced the burden of treatment by decreasing the frequency of clinic visits and the associated transportation costs. They described how this allowed them to maintain their regular lives while adhering to their treatment. In contrast to traditional DOT, HCWs agreed DAT can reduce user burdens and increase autonomy considerably.

... if we go to the centre daily, the fare back and forth will cost us 200 pesos ... it’s difficult if I must go there daily because I can’t work and do anything.

Medication label user, age 18-29 years, male

You have to think economically. If the house is far away, the concern is not just the relationship of having a face-to-face talk, but economically it would burden the client.

NTP medical coordinator, female

Moreover, users found DATs easy to use, describing the experience as trouble-free. They appreciated the specific features of each DAT that helped them stay on their treatment. Medication label users valued the SMS text message reminders, while pillbox users relied on the alarms. One user mentioned that simply seeing the smart pillbox at home served as a reminder to take medication.

I work the night shift, and sometimes the morning shift, sometimes I don’t know the time anymore ... it reminds me that I need to take my medications.

Medication label user, age 30-39 years, female

... it’s good because every time I forget to take medications ... it has an alarm that rings at a quarter to 8 in the morning. So, this pillbox reminds me to take my medications.

Smart pillbox user, age 30-39 years, male

Prior to the implementation, HCWs were concerned that DAT would increase their workload, but once the program was in place, some of them changed their perspective. They reported that DATs added to their tasks but also allowed them to view users’ adherence and helped them adhere to treatment. This enabled HCWs to provide differentiated care to those who missed doses.

At first, I thought it was extra work because you have a lot [of forms] to fill out and with the number of patients we have, a few minutes for each TB patient ...

Midwife, female

I’m able to know immediately with the use of the apps from Everwell who missed their intake, and I would call right after.

Nurse, male

However, both DATs and the adherence platform experienced glitches. Medication label users sometimes received reminders after sending the code or did not receive SMS text message reminders at all. Although a small number of users expressed worry, most users disregarded incorrect reminders, confident that they had taken their medication.

A bit dissatisfied because then I get confused. Of course, at my age, I can be forgetful, but I know in myself that I have taken my medications. When they send that type of text, it makes me think again.

Medication label user, age 40-49 years, female

Pillbox users and HCWs reported pillbox alarm malfunction, and the adherence platform also failed to show user adherence records correctly.

Interviewer: Was there a time when you were not sure what would happen to the pillbox while you were using it?

Participant: Yes, because sometimes it suddenly makes a sound, but sometimes we’ve already taken the medicine but it still makes a sound.

Smart pillbox user, age 50-59 years, female

When the pillbox is opened, sometimes they don’t even turn green.

Midwife, female

In response to the technical challenge, HCWs highlighted the importance of improving the adherence platform by solving the existing glitches and integrating it with the local digital health information system.

Maybe what we mentioned is to integrate the KNCV program with ITIS so that there is less workload for HCWs so that they don’t have to work twice.

Nurse, female

#### Program Design Level

Similarly, users and HCWs shared their positive and negative experiences regarding the program implementation, which had direct implications on the effectiveness and user perception of the intervention. Users generally appreciated the clear explanations and demonstrations provided during their initial DAT consultations with HCWs. HCWs remain supportive throughout the treatment was one of the reasons why users adhered to using DATs and following the treatment.

they will find out what your concern is ... The people are kind. You won’t feel that you are treated like someone who’s sick. You will not feel discriminated that you are sick, that you have TB. They are accommodating ... Ahhh caring.

Medication label user, age 40-49 years, male

Nevertheless, some users reported that the DAT setting did not align with what HCWs had initially informed them during the introduction session, causing confusion. Additionally, individuals working in environments where cell phones were prohibited faced challenges with the use of DAT.

I’m just wondering, the medications are reminded to be taken at 12 midnight in the text message. But the instructions given to me before at the centre are in the morning.

Medication label user, age 30-39 years, female

Cellphones are prohibited in our factory ... Yes, so I get to text during trips.

Medication label user, age 18-29 years, male

Regarding HCWs’ experience, they acknowledged the value of DAT training in understanding its purpose and operation to alleviate misconceptions and misconceived notions of DAT complexity. However, some HCWs expressed concerns about sporadic or insufficient training opportunities, and others mentioned a lack of follow-up or refresher training. In some instances, HCWs were tasked with implementing DAT without adequate training. Hence, HCWs require comprehensive training and adequate resources to effectively implement the program in the future.

I wish there would be another training so that I can really know the program. I was just able to do that by fiddling with my cellphone.

Nurse, male

When she gave it to me, I was not yet trained. There was only one time that she told me to train but that time I couldn’t train either because I was alone.

Midwife, female

HCWs also indicated that there was a shortage of pillboxes in their facilities, leading to the exclusion of persons with TB from the DAT program when they could not use medication labels for whatever reasons.

So, there are times when we can’t enrol the patient to DAT because we don’t have any pillbox available anymore.

Nurse, female

In summary, users and HCWs were satisfied with the result, and they expressed a strong desire for the continuation and expansion of the DAT program in the future.

I wish they continue to give me medicine and always remind me.

Medication label user, age 18-29 years, male

I hope this DAT can used by TB patients all over the Philippines because I think it is really useful.

Nurse, female

#### Community Level

Many users highlighted that TB stigma remained an important issue within their communities. They expressed concerns about discrimination and stigmatization, which often deterred them from disclosing their use of the DAT to persons outside their immediate family.

I told ma’am that there are a lot of gossip mongers here and they might ask why the centre is bringing medicines to me, right?

Smart pillbox user, age 40-49 years, male

Not anymore, but of course there is discrimination, isn’t there? People are not that open about it yet, like, “Oh my gosh, [she has] TB.”

Medication label user, age 30-39 years, female

Nonetheless, both groups stated that DATs and the program design helped mitigate the risk of unintentionally disclosing users’ health conditions through daily visits to TB clinics. Users viewed DAT as a way to protect their privacy and reduce the chances of experiencing stigma. As one medication label user expressed:

Because you stick to texting ... You will not be ashamed to go out as if the stigma you are talking about is already gone ... They don’t know you’re sick ... so you are not immersed in such an issue ... then your treatment is monitored so you come out of the treatment episode quietly.

Sleeve label user, age 40-49 years, male

If there is someone that you know saw you at a TB facility, the perception is you have TB, and they will avoid you. So, by means of texting, and having their medicines daily, their neighbors don’t have to know that they are having checkups with DOTs.

Nurse, female

In this context, stigma not only acts as a factor for users to keep their health condition undisclosed but also serves as an incentive for them to use DAT to avoid revealing their health condition through HCW visits or daily visits to the facilities.

#### Contextual Level

Users and HCWs also shared various contextual factors that influenced their experience with DAT. Medication label users encountered challenges related to the availability of mobile network signals and power in their areas. Users were not able to charge their phones when there was a power outage in the area. These issues prevented them from instantly sending SMS text messages as required. Some service providers in the Philippines also required users to have a positive balance on their SIM to send the code. When users depleted their credit, they were unable to send the code, which then led to receiving reminder SMS text messages from the adherence platform.

It’s just really difficult, for example, when the signal is affected ... What happens is when there is no electricity ... you can’t feedback.

Medication label user, age 40-49 years, male

Interviewer: But you mentioned that there were issues with the network sim card?

Participant: Because I am unable to chat when I don’t have load. That is why I load up whatever amount.

Medication label user, age 18-29 years, male

For HCWs, the challenges posed by the COVID-19 pandemic added to their workload, which, in turn, affected the implementation of the program. Some HCWs were diverted to COVID-19–related tasks, limiting their ability to handle DAT-related tasks.

However, because of COVID-19, there were many cases. My job then was to swab patients, so I wasn’t able to visit the RHU and handle DAT-related tasks.

Medical Technologist, female

HCWs recognized the need for additional resources to address these challenges effectively. They emphasized the importance of improving the network infrastructure to ensure reliable communication for DAT users. Additionally, they called for dedicated staff members to manage and support the DAT program.

I think the system improvement with the network because some clients are complaining that they cannot send a message and they didn’t receive a reply.

Nurse, female

There should be staff handling this program ... I have many programs under me. So really there should be staff who will handle that specific program, correct?

Nurse, female

## Discussion

### Findings

This study illustrates the perceptions and experiences of persons with TB, HCWs, and KIs regarding the use of DATs. By analyzing their responses, we can assess the acceptability of DAT as described by Sekhon et al [19]. As previously highlighted, user and HWC use of the 2 DATs are influenced by overlapping factors. Our analysis also observes DAT-specific strengths and weaknesses, which will be illustrated using a modified social-ecological framework.

At the individual level, the results highlight the critical role of user motivation in achieving successful treatment outcomes. Users demonstrated their commitment to treatment through various means, not solely relying on DAT, HCWs, or family support. This observation aligns with prior research conducted in Pakistan [31]. HCWs noted that DATs assisted users adhere to treatment and fostered a sense of accountability for timely medication intake. This finding is consistent with similar results from Uganda and a meta-analysis [12,22]. However, users with lower technology literacy and financial constraints faced more challenges in using the medication label, potentially leading to exclusion from the program if a smart pillbox was unavailable in the facility. Within this group, acceptability appears to be slightly lower. Increasing the supply of smart pillboxes in the future might assure more users can benefit from the program.

Regarding relationships, DAT significantly alters the user-HCW relationships, even in the context of limited in-person interaction when compared to traditional DOT. Through DAT, persons with TB felt supported by knowing that HCWs were supporting them remotely, even when care delivery had been substantially impacted by COVID-19 restrictions. This finding aligns with similar research conducted in Africa, South Asia, and Southeast Asia contexts [4,12,20,21,32,33]. Technological advancements enabled users and HCWs in the Philippines to stay connected via mobile communication apps such as WhatsApp and Facebook. These communication channels contributed to the enhancement of their relationship, requiring minimal effort on both sides. In this aspect, both groups exhibited a high level of acceptability.

At the technology level, users and HCWs showed high levels of acceptability toward DATs due to their ease of use, assistance in maintaining treatment adherence, and reduced treatment burden. HCWs valued the adherence platform for its ability to assist them in providing adherence support despite the additional workload it entails. However, the interviews revealed that medication label users faced more challenges stemming from external factors such as network issues, power supply, user technology literacy, phone ownership, and SIM card credit. Similar challenges were observed in previous research [20,22]. The smart pillbox on the other hand also encountered alarm malfunction issues. Research in Vietnam and India [13,21] showed that users consider pillbox alarms too loud and inconvenient for travel and might attract unnecessary attention when they visit the clinic to refill their medications. Yet, users from the Philippines did not express these concerns. In addition, the adherence platform sometimes failed to show correct user adherence records. Despite both groups expressing worries when the technology failed and stressing that these glitches need to be addressed, they wanted the DAT program to continue in the future, which shows they have a high degree of acceptance in this domain.

At the community level, TB stigma remained a significant concern causing hesitancy among persons with TB to seek help. The 2 groups stressed how DATs and its operating model in the Philippines effectively protected user privacy, reducing the risk of stigmatization. In contrast to certain other countries, users in this context reported no worries about stigma within their families, given the mandatory requirement for them to be accompanied by a family member or treatment companion during their initial consultation. However, they remained concerned regarding potential stigma at the community and societal level. Users refrained from sharing their use of DAT beyond their immediate family and fearing stigmatization if observed visiting a TB clinic. Research conducted in Ethiopia, Thailand, South Africa, and Zambia found that persons with TB do not want to be seen at TB clinics because of perceived stigmatization [34-37]. As previously outlined, in the Philippines, DATs substantially reduce the frequency of facility visits, from daily to every 2 to 4 weeks, depending on the user’s circumstances, HCW arrangements, and local COVID-19 restrictions of the time. In some instances, community HCWs deliver medication to the person with TB if one lives in remote areas or without a means of transportation. During COVID-19, some local health care facilities also deliver medication to users because of the government’s “no contact policy.” The DAT operating model in the Philippines decreases the frequency of clinic visits, protects users from unintended disclosure, and thus reduces their perceived stigma.

Despite COVID-19 restrictions limiting home visits, users were motivated to adhere to their treatment to avoid being visited by HCWs, which might expose their condition within their community. This could explain why the interview participants demonstrated a high level of acceptance toward DAT, as it can protect them from stigmatization.

Program implementation is closely intertwined with contextual factors. The outbreak of COVID-19 significantly influenced DAT implementation in the Philippines, leading the government to enforce policies aimed at containing the virus’s spread. The pandemic further strained the already burdened health care system in the Philippines. HCWs stationed at research facilities were required to conduct COVID-19 testing and vaccination, diverting their attention and resources from providing differentiated care. This further amplified the workload of HCWs who were enrolling DAT users and managing 2 parallel health information systems without additional staff. Despite these challenges, they acknowledged that DAT enabled real-time monitoring and facilitated prompt follow-up with nonadherent users, ultimately enhancing their efficiency. The consensus among both groups underscored DAT’s capacity to function in regions facing health emergencies and operating in stressed health care systems.

The issue of training remained a persistent concern for HCWs involved with DAT across various settings. Previous research carried out in India revealed that HCWs emphasized the necessity for more DAT training due to the frequent turnover of staff [21]. The phenomenon was also reported by the ASCENT Philippines team. The impact of COVID-19 further heightened matters, with newly recruited HCWs being focused on enrolling persons with TB without sufficient training. This issue is rooted within the health care system, and simply increasing the frequency of DAT training may not fully address the underlying problem. These observations emphasize that the acceptability of DAT is heavily reliant on the local health care system. Expanding the DAT program effectively requires addressing systemic health care issues. Failure to do so may compromise the program’s impact and effectiveness.

### Limitations

This study has successfully captured the perspectives and experiences of persons with TB, HCWs, and KIs in the Philippines who engaged with DATs. It is the first DAT acceptability research conducted in the Southeast Asian context and provides insights on how to improve DAT adoption in the Philippines from a bottom-up approach. However, this study only included individuals who used DATs in their treatment because some persons with TB who demonstrated low levels of treatment adherence or opted out of using DAT declined participation in this research. It was not possible, therefore, to examine the reasons underlying the low levels of adherence or why persons opted out of DATs. Their experiences and perceptions are extremely important for DoH and NTP to formulate a better DAT implementation. Hence, further research is needed to study users who opted out of DATs. This study included only 6 smart pillbox users, so we might risk underreporting issues encountered by smart pillbox users during the treatment.

### Conclusions

In conclusion, this study reveals the experiences, perceptions, and challenges encountered by persons with TB, HCWs, and KIs during the research period. Both groups demonstrated a high level of acceptance and satisfaction toward the positive impact brought by DAT. The challenges encountered underscore the need for ongoing technological development to minimize malfunctions and improve local adoption. The majority of interviewees expressed a desire for the scaling up of DATs in the Philippines.

To achieve this, enhancing the capacity of health facilities through increased staffing, particularly with personnel dedicated to enrollment and user support, is crucial. Regular training can contribute to manageable user monitoring. External factors, such as network availability, SIM card credit, and power supply, need to be fixed to ensure smooth DAT implementation, especially for the medication label, in the future.

Finally, integrating the parallel digital health systems is essential to alleviate the health care workload. Beyond the potential to improve treatment outcomes and reduce loss to follow-up, DAT has demonstrated its ability to enhance user-HCW relationships and prevent unwanted TB treatment disclosure or even stigmatization. Additional efforts are required to enhance the user experience for both persons with TB and HCWs, with the aim of expanding the program to other regions in the Philippines. Although the experiences gained in the Philippines can serve as a reference for other countries in the region considering implementing DAT in the future, it is crucial to recognize that DAT programs need to be tailored to fit within the local health systems and context of each specific region.

## Appendix

Multimedia Appendix 1 Information on the medication label and smart pillbox.

## Abbreviations

ASCENT: Adherence Support Coalition to End TB project
DAT: digital adherence technology
DoH: Department of Health
DOT: directly observed therapy
DS-TB: drug-sensitive tuberculosis
HCW: health care worker
KI: key informant
NTP: National Tuberculosis Control Program
TB: tuberculosis
WHO: World Health Organization

## References

[1] Sagbakken M, Frich JC, Bjune GA, Porter JDH (2013). Ethical aspects of directly observed treatment for tuberculosis: a cross-cultural comparison. BMC Med Ethics.

[2] Yellappa V, Lefèvre P, Battaglioli T, Narayanan D, Van der Stuyft P (2016). Coping with tuberculosis and directly observed treatment: a qualitative study among patients from South India. BMC Health Serv Res.

[3] Cattamanchi A, Crowder R, Kityamuwesi A, Kiwanuka N, Lamunu M, Namale C, Tinka LK, Nakate AS, Ggita J, Turimumahoro P, Babirye D, Oyuku D, Berger C, Tucker A, Patel D, Sammann A, Turyahabwe S, Dowdy D, Katamba A (2021). Digital adherence technology for tuberculosis treatment supervision: a stepped-wedge cluster-randomized trial in Uganda. PLoS Med.

[4] Liu X, Thompson J, Dong H, Sweeney S, Li X, Yuan Y, Wang X, He W, Thomas B, Xu C, Hu D, Vassall A, Huan S, Zhang H, Jiang S, Fielding K, Zhao Y (2023). Digital adherence technologies to improve tuberculosis treatment outcomes in China: a cluster-randomised superiority trial. Lancet Glob Health.

[5] Manyazewal T, Woldeamanuel Y, Holland DP, Fekadu A, Marconi VC (2022). Effectiveness of a digital medication event reminder and monitor device for patients with tuberculosis (SELFTB): a multicenter randomized controlled trial. BMC Med.

[6] Boutilier JJ, Yoeli E, Rathauser J, Owiti P, Subbaraman R, Jónasson JO (2022). Can digital adherence technologies reduce inequity in tuberculosis treatment success? Evidence from a randomised controlled trial. BMJ Glob Health.

[7] Wei X, Hicks JP, Zhang Z, Haldane V, Pasang P, Li L, Yin T, Zhang B, Li Y, Pan Q, Liu X, Walley J, Hu J (2024). Effectiveness of a comprehensive package based on electronic medication monitors at improving treatment outcomes among tuberculosis patients in Tibet: a multicentre randomised controlled trial. Lancet.

[8] Acosta J, Flores P, Alarcón M, Grande-Ortiz M, Moreno-Exebio L, Puyen Z (2022). A randomised controlled trial to evaluate a medication monitoring system for TB treatment. Int J Tuberc Lung Dis.

[9] Velen K, Nguyen TA, Pham CD, Le HT, Nguyen HB, Dao BT, Nguyen TV, Nguyen NT, Nguyen NV, Fox GJ (2023). The effect of medication event reminder monitoring on treatment adherence of TB patients. Int J Tuberc Lung Dis.

[10] Metcalfe JZ, O'Donnell MR, Bangsberg DR (2015). Moving beyond directly observed therapy for tuberculosis. PLoS Med.

[11] Subbaraman R, de Mondesert L, Musiimenta A, Pai M, Mayer KH, Thomas BE, Haberer J (2018). Digital adherence technologies for the management of tuberculosis therapy: mapping the landscape and research priorities. BMJ Glob Health.

[12] Musiimenta A, Tumuhimbise W, Mugaba A, Muzoora C, Armstrong-Hough M, Bangsberg D, Davis JL, Haberer JE (2019). Digital monitoring technologies could enhance tuberculosis medication adherence in Uganda: mixed methods study. J Clin Tuberc Other Mycobact Dis.

[13] Drabarek D, Anh NT, Nhung NV, Hoa NB, Fox GJ, Bernays S (2019). Implementation of Medication Event Reminder Monitors among patients diagnosed with drug susceptible tuberculosis in rural Viet Nam: a qualitative study. PLoS One.

[14] Thekkur P, Kumar AN, Chinnakali P, Selvaraju S, Bairy R, Singh AR, Nirgude A, Selvaraj K, Venugopal V, Shastri S (2019). Outcomes and implementation challenges of using daily treatment regimens with an innovative adherence support tool among HIV-infected tuberculosis patients in Karnataka, India: a mixed-methods study. Glob Health Action.

[15] Liu X, Blaschke T, Thomas B, De Geest S, Jiang S, Gao Y, Li X, Buono EW, Buchanan S, Zhang Z, Huan S (2017). Usability of a medication event reminder monitor system (MERM) by providers and patients to improve adherence in the management of tuberculosis. Int J Environ Res Public Health.

[16] Bediang G, Stoll B, Elia N, Abena JL, Geissbuhler A (2018). SMS reminders to improve adherence and cure of tuberculosis patients in Cameroon (TB-SMS Cameroon): a randomised controlled trial. BMC Public Health.

[17] Mohamed MS, Zary M, Kafie C, Chilala CI, Bahukudumbi S, Foster N, Gore G, Fielding K, Subbaraman R (2024). The impact of digital adherence technologies on health outcomes in tuberculosis: a systematic review and meta-analysis. medRxiv (forthcoming).

[18] Jerene D, Levy J, van Kalmthout K, van Rest J, McQuaid CF, Quaife M, Charalambous S, Gamazina K, Garfin AMC, Mleoh L, Terleieva Y, Bogdanov A, Maraba N, Fielding K (2023). Effectiveness of digital adherence technologies in improving tuberculosis treatment outcomes in four countries: a pragmatic cluster randomised trial protocol. BMJ Open.

[19] Sekhon M, Cartwright M, Francis JJ (2017). Acceptability of healthcare interventions: an overview of reviews and development of a theoretical framework. BMC Health Serv Res.

[20] Thomas BE, Kumar JV, Onongaya C, Bhatt SN, Galivanche A, Periyasamy M, Chiranjeevi M, Khandewale AS, Ramachandran G, Shah D, Haberer JE, Mayer KH, Subbaraman R (2020). Explaining differences in the acceptability of 99DOTS, a cell phone-based strategy for monitoring adherence to tuberculosis medications: qualitative study of patients and health care providers. JMIR Mhealth Uhealth.

[21] Thomas BE, Kumar JV, Periyasamy M, Khandewale AS, Hephzibah Mercy J, Raj EM, Kokila S, Walgude AS, Gaurkhede GR, Kumbhar JD, Ovung S, Paul M, Rajkumar BS, Subbaraman R (2021). Acceptability of the medication event reminder monitor for promoting adherence to multidrug-resistant tuberculosis therapy in two Indian cities: qualitative study of patients and health care providers. J Med Internet Res.

[22] Guzman K, Crowder R, Leddy A, Maraba N, Jennings L, Ahmed S, Sultana S, Onjare B, Shilugu L, Alacapa J, Levy J, Katamba A, Kityamuwesi A, Bogdanov A, Gamazina K, Cattamanchi A, Khan A (2023). Acceptability and feasibility of digital adherence technologies for drug-susceptible tuberculosis treatment supervision: a meta-analysis of implementation feedback. PLOS Digit Health.

[23] (2023). Tuberculosis profile: Philippines. World Health Organization.

[24] Romualdez AG (2007). TB-DOTS in the Philippines: impact of decentralization and health sector reform. Bull World Health Organ.

[25] (2021). NTP Manual of Procedures 6th Edition. National Tuberculosis Control Program-Department of Health.

[26] Braun V, Clarke V (2006). Using thematic analysis in psychology. Qual Res Psychol.

[27] Houston S (2001). Beyond social constructionism: critical realism and social work. Br J Social Work.

[28] Sazzad HMS, McCredie L, Treloar C, Lloyd AR, Lafferty L (2020). Violence and hepatitis C transmission in prison—a modified social ecological model. PLoS One.

[29] McLeroy KR, Bibeau D, Steckler A, Glanz K (1988). An ecological perspective on health promotion programs. Health Educ Q.

[30] Rhodes T, Singer M, Bourgois P, Friedman SR, Strathdee SA (2005). The social structural production of HIV risk among injecting drug users. Soc Sci Med.

[31] Mohammed S, Glennerster R, Khan A (2016). Impact of a daily SMS medication reminder system on tuberculosis treatment outcomes: a randomized controlled trial. PLoS One.

[32] Hoffman JA, Cunningham JR, Suleh AJ, Sundsmo A, Dekker D, Vago F, Munly K, Igonya EK, Hunt-Glassman J (2010). Mobile direct observation treatment for tuberculosis patients: a technical feasibility pilot using mobile phones in Nairobi, Kenya. Am J Prev Med.

[33] Leddy A, Ggita J, Berger C, Kityamuwesi A, Sanyu AN, Tinka LK, Crowder R, Turyahabwe S, Katamba A, Cattamanchi A (2023). Barriers and facilitators to implementing a digital adherence technology for tuberculosis treatment supervision in Uganda: qualitative study. J Med Internet Res.

[34] Demissie M, Getahun H, Lindtjørn B (2003). Community tuberculosis care through "TB clubs" in rural North Ethiopia. Soc Sci Med.

[35] Chaychoowong K, Watson R, Barrett D (2023). Perceptions of stigma among pulmonary tuberculosis patients in Thailand, and the links to delays in accessing healthcare. J Infect Prev.

[36] DeSanto D, Velen K, Lessells R, Makgopa S, Gumede D, Fielding K, Grant AD, Charalambous S, Chetty-Makkan CM (2023). A qualitative exploration into the presence of TB stigmatization across three districts in South Africa. BMC Public Health.

[37] Cremers AL, de Laat MM, Kapata N, Gerrets R, Klipstein-Grobusch K, Grobusch MP (2015). Assessing the consequences of stigma for tuberculosis patients in urban Zambia. PLoS One.

