# Supplementary material for: Digital Adherence Technologies and Differentiated Care for Tuberculosis Treatment and Their Acceptability Among Persons With Tuberculosis, Health Care Workers, and Key Informants in the Philippines: Qualitative Interview Study
Source: JMIR Hum Factors. 2024 Jul 23;11:e54117. doi: 10.2196/54117 (PMC11303897; doi:10.2196/54117)

**Multimedia Appendix 1.** Information on the medication label and smart pillbox.

### Medication Label

A customized sticker is attached to the standard TB medication blister pack. Each sticker contains a variable three-digit code and a standardized toll-free number. After taking their daily medication, patients are required to send the code to the toll-free number to log their dose.

The adherence platform records the patient's medication intake and marks their adherence record as green, and returns a congratulatory SMS to the patient. If a patient forgets to send the code within a specified timeframe, they will receive a reminder SMS prompting them to take their medication and log their dose before midnight.. If the patient still fails to send the code before the end of the day at midnight, the day is marked red in their adherence calendar and a reminder SMS is sent at 8:00 the following day, notifying them of the missed dose and reminding them to not miss their next dose.


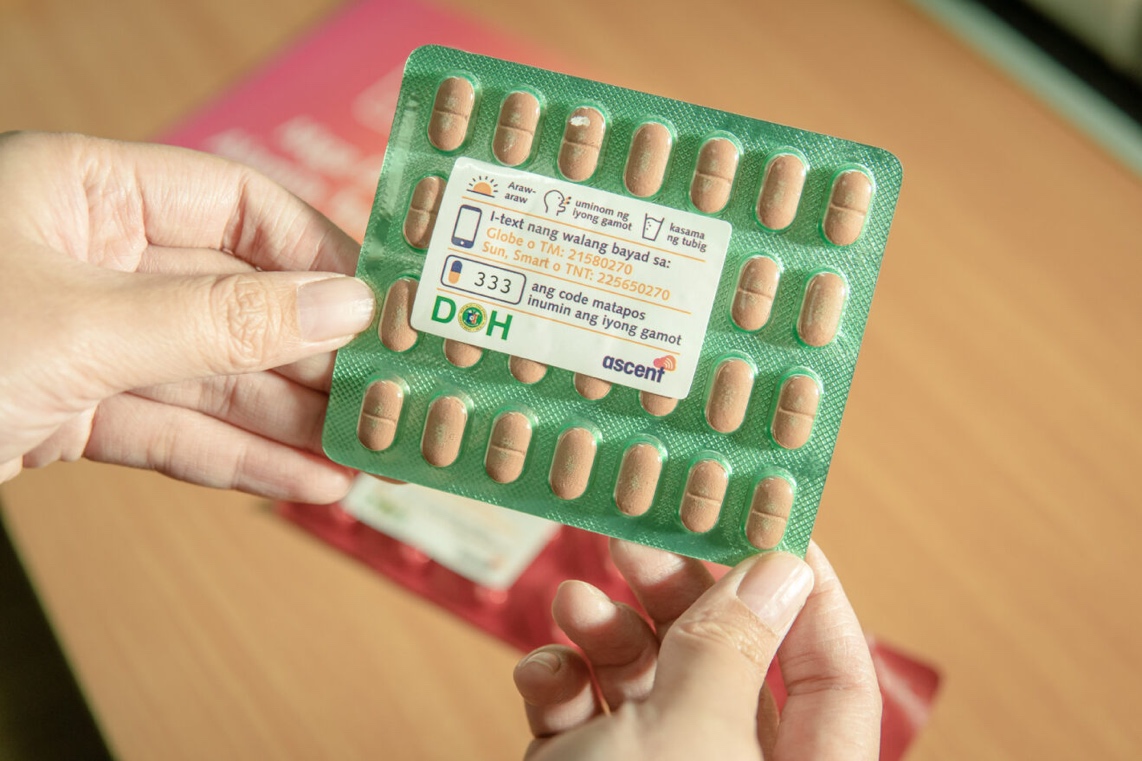


### Smart Pillbox

A pillbox was provided to the patient to store their medication. The box has a capacity of up to four weeks of DS-TB medication. Patients are required to open the box daily to take their medication. The box contains a module outfitted with sensor and eSIM-enabled microprocessor. When the box is opened, the module senses the action and syncs the timestamped dosing information with to the adherence platform via the mobile network. Patient had the choice to opt-in for enabling the alarm function on the box with a time agreed by patient and HCW. If a patient fails to open the box within a specified timeframe, their adherence record will mark as red in the adherence platform and it will trigger an SMS reminder to the patient’s registered phone number. In cases of unstable network connection, the box records the opening and transmits the timestamped data once the network is available again.

 
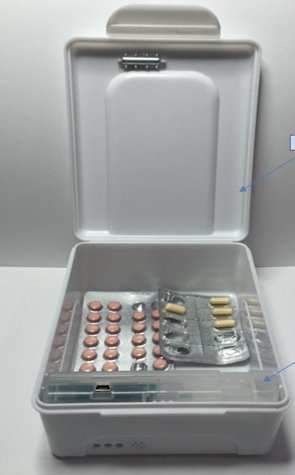

Supplement: Multimedia Appendix 1 [file humanfactors_v11i1e54117_app1.docx]
